# Oncolytic Viruses for Multiple Myeloma Therapy

**DOI:** 10.3390/cancers10060198

**Published:** 2018-06-14

**Authors:** Christine M. Calton, Kevin R. Kelly, Faiz Anwer, Jennifer S. Carew, Steffan T. Nawrocki

**Affiliations:** 1 Division of Translational and Regenerative Medicine, Department of Medicine and The University of Arizona Cancer Center, Tucson, AZ 85724, USA; calton@email.arizona.edu (C.M.C.); jcarew@email.arizona.edu (J.S.C.); 2 Jane Anne Nohl Division of Hematology and Center for the Study of Blood Diseases, University of Southern California Norris Comprehensive Cancer Center, Los Angeles, CA 90033, USA; kevin.kelly@med.usc.edu; 3 Division of Hematology and Oncology, University of Arizona Cancer Center, Tucson, AZ 85724, USA; anwerf@email.arizona.edu

**Keywords:** multiple myeloma, oncolytic virotherapy, reovirus, measles virus, vesicular stomatitis virus, vaccinia virus, myxoma virus, adenovirus

## Abstract

Although recent treatment advances have improved outcomes for patients with multiple myeloma (MM), the disease frequently becomes refractory to current therapies. MM thus remains incurable for most patients and new therapies are urgently needed. Oncolytic viruses are a promising new class of therapeutics that provide tumor-targeted therapy by specifically infecting and replicating within cancerous cells. Oncolytic therapy yields results from both direct killing of malignant cells and induction of an anti-tumor immune response. In this review, we will describe oncolytic viruses that are being tested for MM therapy with a focus on those agents that have advanced into clinical trials.

## 1. Introduction

Multiple myeloma (MM) is a malignancy of clonal plasma cells. It is the second most common hematological cancer in the United States with an estimated 30,280 newly diagnosed cases and 12,590 deaths in 2017. MM primarily affects elderly patients with a median age at diagnosis of 69 years in the United States. It currently accounts for 2.1% of all cancer-related deaths [1].

MM belongs to a group of conditions collectively known as monoclonal gammopathies, which are characterized by abnormally high levels of monoclonal immunoglobulin protein and clonal plasma cells in the bone marrow [2]. The most common of these disorders is monoclonal gammopathy of undetermined significance (MGUS). MGUS itself is asymptomatic, but can progress to active MM. The risk of progression is approximately 1% per year [3]. An intermediary stage known as smoldering myeloma may occur during progression from MGUS to MM [4]. Active MM is characterized by hypercalcemia, renal insufficiency, anemia, and bone lesions [2]. 

Current treatment options for MM are pharmacological therapy and autologous hematopoietic stem cell transplant (AHSCT). Three classes of drugs are primarily used for MM therapy: immunomodulatory agents, proteasome inhibitors and monoclonal antibodies. The immunomodulatory drugs lenalidomide and pomalidomide are thalidomide-derivatives that are thought to alter the tumor microenvironment in a manner that promotes MM cell killing by the immune system in addition to having anti-proliferative and anti-angiogenic effects [5]. Proteasome inhibitors, such as bortezomib and carfilzomib, induce apoptotic death by preventing the normal turnover of cellular proteins. Cells that produce large quantities of protein, such as malignant plasma cells, are particularly sensitive to the effects of proteasome inhibition [6]. Combination therapy with these drugs has significantly improved patient survival time [7]. Lenalidomide and bortezomib in combination with the corticosteroid dexamethasone is currently the treatment of choice for initial presentation of MM. AHSCT, in which a patient’s hematopoietic stem cells are collected, purged of malignant cells, and then reinfused into the patient, has become less common due to improvements in pharmacological therapy. When AHSCT is used today, it is often combined with immunomodulatory and proteasome inhibitor treatments [2]. In addition to these strategies, two monoclonal antibodies, elotuzumab (targeting CS1/SLAMF7) and daratumumab (targeting CD38), have recently been approved for MM therapy [8].

The introduction of targeted chemotherapeutics and AHSCT has significantly increased survival time for MM patients. However, none of the therapies are curative and most patients will eventually become refractory to current treatment options. Therapeutics with new mechanisms of action are therefore needed to treat drug-resistant MM. Oncolytic viruses have emerged as a promising new class of agents with great potential for the treatment of MM.

## 2. Viral Oncolytics for Multiple Myeloma

**Figure 1:**
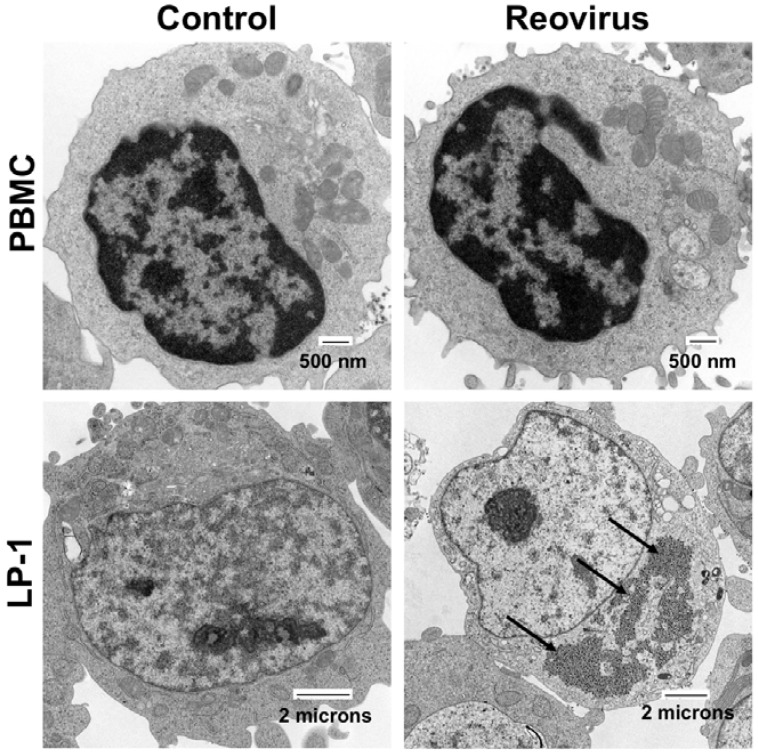
Reovirus (RV) selectively replicates in multiple myeloma cells. Normal peripheral blood mononuclear cells (PBMCs) and LP-1 MM cells were treated with 30 plaque forming units/cell RV for 48 h. RV was detected by electron microscopy. Arrows denote RV.

Oncolytic viruses specifically replicate in and kill tumor cells (Figure 1). All oncolytic virotherapies are derived from naturally occurring viruses. However, many of the viruses being developed for oncolytic therapy have been modified to increase their specificity for cancer cells or enhance their ability to promote tumor clearance. MM has several features that make it an ideal target for oncolytic virotherapy. These include mutations in signaling pathways that render MM more sensitive to viral infection and overexpression of cell surface proteins that are commonly used as viral entry receptors [9,10,11]. In the following section, we will describe oncolytic viruses that are currently being tested as therapeutics in MM (Table 1). Most research has focused on developing oncolytics that can be directly administered to MM patients. However, some viruses are also being explored for use as purging agents in AHSCT.

### 2.1. Reovirus

Mammalian reoviruses (RV) belong to the *Reoviridae* family of viruses. The genome consists of 10 double-stranded RNA segments contained within two concentric protein shells [12]. RV infects the respiratory and enteric tracts and is generally benign in humans. Most of the circulating antibodies against RV in adults are likely from childhood exposure to the virus [13,14,15]. Three different RV serotypes have been identified based on hemagglutination and neutralization assays [16]. Currently, the only RV under development for oncolytic therapy is the human type 3 Dearing strain. The recent development of a reverse genetics system for RV should make genetic manipulations more feasible in the future [17].

RV entry into cells requires sequential binding to different host receptors. Initial attachment is mediated by extracellular sialic acid followed by engagement of junctional adhesion molecule A (JAM-A) [18,19,20]. JAM-A expression in the epithelium and endothelium helps maintain tight junction integrity. It is also expressed on hematopoietic cells, where it regulates leukocyte transmigration [21]. Endothelial expression of JAM-A is required for RV egress from the bloodstream to other sites in the body, which has important implications for the use of RV as an oncolytic therapy [22]. Following endocytosis of the virion, the outer shell of the RV capsid is degraded by lysosomal cathepsins B and L, which in turn frees the inner core to enter the cytoplasm where viral replication occurs [23]. 

**Figure 2:**
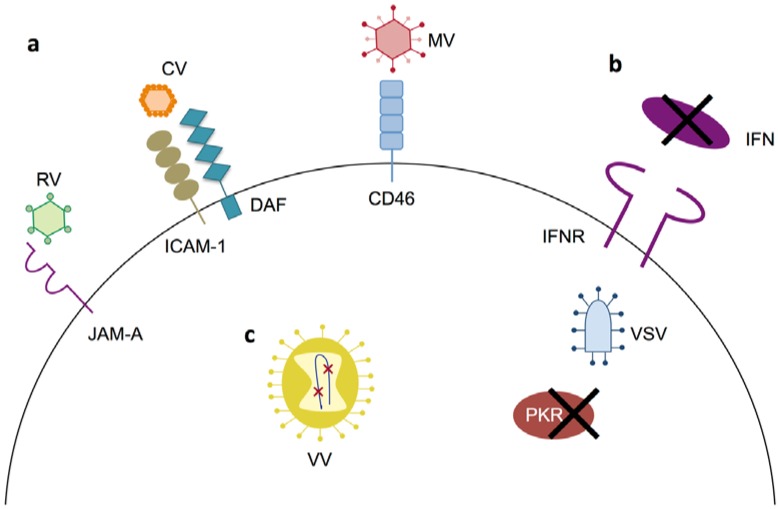
Primary mechanisms of tumor specificity for oncolytic virotherapies in multiple myeloma. (**a**) For RV, CV and MV tumor specificity is dictated by their respective receptors, each of which is overexpressed in MM; (**b**) Deficiencies in IFN signaling and PKR activity, which are common in MM, provide tumor specificity for VSV; (**c**) VV tumor specificity is driven by engineered deletions in the vaccinia genome that eliminate genes essential for viral replication in normal cells. Additional mechanisms of tumor specificity exist for many of the viruses depicted; see the text for details.

In normal cells, viral transcripts generated during replication can induce phosphorylation and activation of protein kinase R (PKR), a serine/threonine kinase that acts as a sensor of viral infection [24]. Activated PKR phosphorylates the α subunit of elongation initiation factor 2, which results in shutdown of most cellular translation [24]. Thus, in healthy tissue RV infection is aborted as no new viral particles can be made. However, in cells with constitutively active Ras signaling PKR activation is blocked and protein translation and viral particle formation can proceed uninhibited [25]. The dependency on Ras signaling has made RV an attractive candidate for oncolytic therapy [26] as greater than 30% of all human tumors contain Ras-activating mutations [27]. However, recent studies have suggested that mechanisms other than Ras activation may sensitize some cell types to RV killing [28,29]. For example, Ras activation status does not confer susceptibility to RV in MM. Instead, sensitivity to RV positively correlates with JAM-A expression, which is upregulated in MGUS and MM patient specimens (Figure 2) [30]. 

Several pre-clinical studies have demonstrated the efficacy of RV therapy in MM. RV kills MM cell lines and primary patient cells by inducing endoplasmic reticulum (ER) stress, apoptosis and autophagy and significantly reduces tumor burden in mouse xenograft models of MM [31,32]. Furthermore, combination treatment with RV and bortezomib results in a significantly greater reduction of disease than either single agent treatment [31]. Combining RV with the immunomodulatory agent lenalidomide also enhances MM cell killing [33]. Given that most MM patients become refractory to conventional therapies, Kelly et al. tested the efficacy of RV treatment in bortezomib-resistant MM. Bortezomib-resistant MM cell lines and samples from refractory patients were significantly more sensitive to killing by RV than bortezomib-sensitive controls. Increased sensitivity to RV correlated with JAM-A expression, which is significantly upregulated in the relapsed/refractory patient population compared to newly diagnosed patients [31]. These results indicate that RV may be a particularly effective therapy for patients with relapsed/refractory MM. Given this finding, it is interesting to note that histone deacetylase (HDAC) inhibitors have been reported to enhance RV killing of MM via upregulation of JAM-A [34]. This suggests that pharmacological agents that induce JAM-A expression may be useful for augmenting RV therapy. RV has also been investigated as a purging agent for AHSCT, to remove contaminating CD138^+^ cells prior to reinfusion [35,36].

A phase I single agent trial of RV in patients with relapsed MM was reported in 2014 (Table 2) [37]. The treatment was well tolerated, and viral RNA was detected in most of the specimens. However, few virus-containing cells stained positive for caspase 3, indicating a lack of oncolytic killing and the best responses observed were stable disease. These results suggest that RV is not an effective monotherapy in MM. There are three active phase I trials combining RV with approved MM therapeutics: the immunomodulatory agents lenalidomide or pomalidomide (NCT03015922), bortezomib plus dexamethasone (NCT02514382), and carfilzomib plus dexamethasone (NCT02101944). In addition, a clinical trial investigating RV with anti-PD-1 antibody therapy is currently being developed by our group based on promising preclinical data [38].

### 2.2. Measles Virus

*Measles virus* (MV) is an enveloped negative-sense single-stranded RNA virus in the family *Paramyxoviridae*. It is the etiological agent of measles, a childhood disease of the respiratory tract. Today, all vaccines are based on the lab-attenuated Edmonston strain that was first isolated in 1954 and has a long history of safe use in humans [39]. The Edmonston strain is also the basis for measles oncolytics being tested with MM.

MV enters host cells by binding to receptors on the cell surface. This binding induces conformational changes that allow for fusion of the viral envelope with the plasma membrane and delivers the measles genome into the cytoplasm, where replication occurs [39]. Wild type measles strains utilize two cell-type specific receptors: the signaling lymphocyte activation molecule (SLAM) on dendritic cells and macrophages [40] and nectin 4 on epithelial cells [41]. However, the Edmonston strain enters cells via binding to CD46 [42], a type I integral membrane protein found on the surface of all somatic cells [43] and overexpressed in many cancers, including MM [44,45]. Tumor specificity is largely driven by the Edmonston strain’s tropism for CD46 (Figure 2). Increased CD46 levels on CD138^+^ MM cells positively correlate with MV cellular entry, replication, and cytopathic effects. Importantly, normal BM cells from myeloma patients do not overexpress CD46 and are not susceptible to MV killing [44]. The cytopathic effects of MV infection are primarily mediated by virus-induced intercellular fusion, which allows infection to spread without the production of new progeny virus [46]. Anderson et al. found that high CD46 receptor densities are required for MV-induced intercellular fusion, which greatly enhanced cell killing [47]. Other factors that likely contribute to the tumor specificity of MV are impairment of both PKR activation and interferon (IFN) signaling in cancer cells [45].

The oncolytic potential of MV in hematological malignancies was first recognized in the 1970s, when a series of case studies documented regression of leukemia, Burkitt’s lymphoma and Hodgkin’s disease in patients with concurrent measles infections [48,49,50,51]. The first preclinical study in MM showed that the Edmonston strain of MV selectively replicates in and effectively kills myeloma cells from both established tissue culture lines and primary patient samples. Significant reductions in tumor volumes were also observed in mouse xenograft models following treatment with either intratumoral or intravenous injection of MV [52]. Reverse genetics [53] have been used to create versions of MV that can specifically target MM via receptors other than CD46 [54,55,56], evade innate immune defenses to prevent viral clearance [57], or express reporter genes for monitoring efficacy of treatment in patients [58,59,60]. The best characterized of these systems is a variant of the Edmonston strain that has been engineered to express the human sodium iodide symporter (MV-NIS) [58]. NIS is a membrane ion channel expressed by thyroid follicular cells to import iodine for use in thyroid hormone synthesis. This results in highly concentrated levels of iodine within the thyroid (20–40 times greater than plasma levels) [61]. The ability of NIS to concentrate iodine at such high levels allows for the use of radioactive iodine in imaging (I^123^) and treatment (β-emitting I^131^) of malignancies expressing high levels of NIS [62]. In a mouse xenograft model of MV-resistant MM, treatment with MV-NIS plus I^131^ radiotherapy significantly improved tumor regression compared to MV-NIS treatment alone [58]. 

Based on the preclinical results, a phase I/II trial (NCT00450814) was initiated to test MV-NIS treatment in patients with recurrent or refractory myeloma in combination with or without cyclophosphamide (Table 2), an alkylating agent that is approved for myeloma therapy [2]. Phase I of the study, which did not include co-treatment with I^131^, found that MV-NIS was reasonably well tolerated at high doses and preferentially infected CD138^+^ cells over CD138^−^ cells. One patient attained complete remission lasting nine months and four others experienced transient drops in free light chain serum levels [63]. Two other clinical trials for MV are currently active. NCT02192775 is a phase II trial combining MV-NIS with cyclophosphamide in relapsed/refractory MM; no results have yet been published. NCT03456908 is new phase I trial in patients with recurrent/refractory MM. It will test whether the usage of PET scans provides superior imaging to SPECT scans for monitoring NIS expression following MV-NIS treatment. Subjects will be recruited from eligible patients already enrolled in NCT00450814.

### 2.3. Vesicular Stomatitis Virus

*Vesicular stomatitis virus* (VSV), a member of the family *Rhabdoviridae*, is an enveloped negative-sense single-stranded RNA virus. VSV commonly infects cattle, horses, and pigs, where it causes the characteristic vesicular lesions from which its name is derived. Human infections, which can result in acute flu-like disease, are limited to those in direct contact with infected animals [64]. The lack of a human reservoir for VSV makes it an attractive candidate for oncolytic therapy.

VSV enters the cell via receptor-mediated endocytosis. Inside early endosomes, the viral envelope fuses with the endosomal membrane, releasing the nucleocapsid into the cytoplasm, where replication occurs [65]. VSV binding to host cells is mediated by the low density lipoprotein family of receptors (LDLRs) [66]. Members of the LDLR family are ubiquitously expressed throughout the body [67], which likely explains the broad cellular tropism of VSV. Although VSV can enter a variety of cell types, productive infection in humans is usually prevented by the exquisite sensitivity of VSV to the human innate immune response. In normal cells, PKR activation and type-I IFN production potently inhibit VSV replication. However, VSV readily replicates in a variety of tumor types with defects in PKR activation or type-I IFN signaling (Figure 2) [68,69]. Stojdl et al. created a further attenuated strain by deleting methionine 51 in the VSV matrix protein (VSV∆51). This mutation ablates the virus’ ability to block type-I IFN production, thus preventing infection of healthy cells, while maintaining a high degree of lytic activity in IFN-deficient tumor cells [70]. VSV∆51 is the basis for most of the oncolytic VSV therapeutics currently being developed.

Preclinical studies have demonstrated the effectiveness of VSV in both in vitro and in vivo models of MM. VSV was engineered to express NIS, allowing for imaging and treatment with radioactive iodine as described above for MV [71]. Treatment with the resulting strain, VSV-NIS, reduced tumor volumes in a syngeneic, immunocompetent mouse model of MM. Radiotherapy with isotope I^131^ augmented this reduction. To further enhance oncolytic activity and improve safety, the IFN-β gene was inserted into the VSV genome [72,73]. For mouse studies, the murine IFN-β gene was utilized, as human IFN-β is not functional in mice. VSV-mIFN-β-NIS treatment significantly improved tumor responses and prolonged survival compared to treatment with control VSV [72,73]. Notably, although bortezomib antagonizes VSV replication in MM cell lines, combining the two therapies in vivo led to a greater reduction of tumor volumes than VSV alone [74]. 

The promising preclinical data has led to the establishment of a phase I trial for VSV (Table 2). This trial (NCT03017820) will establish dosage and toxicities for VSV-hIFN-β-NIS in patients with relapsed/refractory MM. A comparison of PET and SPECT scans for imaging of VSV-hIFN-β-NIS infected lesions is also being tested in the same trial (NCT00450814) that is testing those imaging systems for use with MV-NIS (see above).

### 2.4. Other Oncolytic Viruses

The viruses described below, while not yet in clinical trials for MM, have shown promise in preclinical studies.

#### 2.4.1. Poxviruses

The *Poxviridae* are a family of large enveloped double-stranded DNA viruses [75]. Members of this family infect a variety of arthropods and animals including humans. Poxviruses can enter and replicate within a variety of cell types and their cytoplasmic replication lessens the chances of recombination into the host genome. Additionally, their large DNA genomes make poxviruses well suited to genetic manipulation by allowing insertion of exogenous genes that could modulate cell killing.

Vaccinia virus (VV) is derived from the original cowpox or horsepox virus that Edward Jenner first used to vaccinate against smallpox in the eighteenth century [76]. Live vaccinia vaccines have been administered by the World Health Organization to over 200 million people worldwide, giving the virus an excellent history of safety in humans [77]. Wild type VV is not inherently oncotropic. Therefore, viral genes that are essential for VV replication in normal cells, such as thymidine kinase (TK) and vaccinia growth factor (VGF), must be deleted to confer tumor specificity (Figure 2) [78,79]. The first reported use of vaccinia as an oncolytic therapy for MM occurred in 1987, when a patient with IgA MM saw a significant decrease in IgA levels and an increase in natural killer cell activity after intravenous treatment with VV [80]. Subsequent in vitro studies utilizing a strain double deleted for TK and VGF showed that MM cell lines are susceptible to killing by VV [81,82]. Viral replication was observed in primary MM cells, but not in normal peripheral blood mononuclear cells (PBMCs) [81]. The double deleted strain also reduced tumor volume and increased survival time in a mouse xenograft model of MM [81]. Recently, Lei et al. used a TK-deleted VV strain to overexpress two anti-tumor factors, miR-34a and Smac, which are frequently dysregulated in MM. Combined treatment with VV-miR-34a and VV-Smac showed increased efficacy against MM both in vitro and in vivo when compared to treatment with the parental virus, VV-miR-34a, or VV-Smac individually [83].

Myxoma virus (MYXV) has a strict tropism for rabbits and hares [75] and therefore does not cause significant disease in humans. Although it cannot infect normal human cells, MYXV has been shown to productively infect a variety of cancer types [84]. In MM cell lines, MYXV induces efficient cell killing that is dependent upon caspase-8 mediated apoptosis and by inhibiting ATF4 expression during the unfolded protein response [85,86,87]. Killing occurs in both bortezomib-sensitive and bortezomib-resistant cells [87], suggesting that MYXV may be useful for treatment of refractory MM. In a syngeneic mouse model of MM, MYXV treatment significantly reduced tumor burden and prolonged survival time, while leaving the healthy bone marrow niche intact [88]. Notably, MYXV-induced cell death is rapid and occurs independent of viral replication [85]. Due to its rapid killing time in MM, MYXV has been proposed as an efficient purging mechanism for AHSCT. To this end, pretreatment with MYXV prevented engraftment of human MM cell lines in a mouse xenograft model. Furthermore, MYXV eliminated CD138^+^ cells from MM patient BM samples within 24 h of treatment [85]. Recent work has shown that MYXV treatment can reduce graft-versus-host disease (GVHD), while promoting graft-versus-tumor responses. MYXV infection of T lymphocytes, which are frequent drivers of GVHD, inhibited their proliferation and activity [89]. In contrast, MYXV treatment of mouse BM-derived neutrophils lead to increased neutrophil activation and more efficient killing of mouse MM cells [90].

#### 2.4.2. *Coxsackie Virus*

*Coxsackie viruses* (CV) are non-enveloped positive-sense single-stranded RNA viruses in the family *Picornaviridae* [91]. At least 29 strains of CV have been identified [91]. CVA21, which causes mild respiratory disease in humans [91], is the primary isolate being developed for use in oncolytic therapy. A preclinical study demonstrated that CVA21 effectively killed MM cell lines as well as CD138^+^ cells from patients with MGUS, newly diagnosed MM and relapsed MM [92]. MM cells were shown to have increased expression of both ICAM-1 and DAF [92], which form a complex to facilitate CVA21 cellular entry [93]. Intriguingly, intratumoral injection of CVA21 RNA alone was sufficient to reduce tumor volumes and increase survival time in a mouse xenograft model of MM [94]. One barrier to using CVA21 in oncolytic therapy is its potential to cause myositis in patients [95]. To circumvent this problem, muscle-specific miRNAs were inserted into the 3′ untranslated region of the CVA21 genome [96]. Intratumoral injection of CVA21 into mice bearing MM xenografts reduced tumor volumes and prolonged survival. Importantly, while mice treated with wild type CVA21 experienced significant muscle inflammation and necrosis, those treated with the muscle-specific miRNA CVA21 exhibited no such pathology [96].

#### 2.4.3. Adenoviruses

*Adenoviridae* is a family of non-enveloped double-stranded DNA viruses that infect a broad range of vertebrate hosts [97]. In humans, 57 different adenovirus (AdV) serotypes have been identified [98]. AdVs are highly amenable to genetic manipulation and have been investigated extensively as vectors for gene therapy [98] and oncolytics, primarily in solid tumors [99]. Preclinical studies have identified several AdV serotypes with oncolytic activity in MM including species B AdV5 [100], the best studied AdV oncolytic in solid tumors [99], and several relatively uncharacterized species D AdV [101]. Improved oncolytic activity against MM cells has been reported with AdV that were engineered to express a CD40 ligand transgene [102] or with the herpes simplex virus TK gene in conjunction with ganciclovir treatment [103].

## 3. Strategies for Improving Oncolytic Virus Efficacy

### 3.1. Carrier Cells

Several of the oncolytic viruses being developed for MM treatment are human pathogens. Therefore, many patients have pre-existing immunity to them either through environmental exposure or vaccination. This presents a challenge for successful therapy as it shortens the length of time that virotherapies can function before they are cleared by the immune system. Even viruses that are not endemic to humans, such as VSV and MYXV, will eventually induce anti-viral responses. Several strategies are being developed to reduce anti-viral responses during oncolytic therapy in MM including serotype switching [104], chemical shielding with polymers [105,106], or the use of immunosuppressive drugs. One novel strategy is the use of carrier cells to deliver viruses directly to tumor targets. Liu et al. used lethally irradiated MM cells to deliver MV-NIS to tumor sites in a mouse model of disseminated myeloma. Treatment with MV-loaded MM cells prolonged survival rates even in the presence of anti-MV neutralizing antibodies [107]. Furthermore, in the case of RV, it has been shown that PBMCs transiently carry the virus after infusion, protecting it from neutralization [108]. These results suggest that carrier cells may be a useful method to boost oncolytic virus longevity and efficacy in MM treatment.

### 3.2. Combination Therapy

Given that oncolytic viruses stimulate a robust immune response, combination therapy with immunomodulatory drugs is an approach to heighten the anti-tumor immune response. RV is currently under clinical investigation in combination with lenalidomide or pomalidomide, immunomodulatory agents that are commonly used in MM therapy (Table 2). The alkylating agent cyclophosphamide also is attractive for combination therapy with oncolytic viruses because of its potential to suppress anti-viral immune responses. Cyclophosphamide is an approved therapeutic for MM and is also used for immunosuppressive treatment in autoimmune disease and transplantation [109]. Combination treatment with cyclophosphamide improves the efficacy of oncolytic viruses by downregulating anti-viral immune responses [110,111,112,113]. It has also been shown to specifically reduce production of anti-MV and anti-VSV neutralizing antibodies [114]. Given that the majority of the population already has immunity against MV [115], cyclophosphamide could be a particularly valuable tool for measles oncolytic therapy. Combination therapy with cyclophosphamide is currently being used in clinical trials for both MV and VSV (Table 2).

Immune checkpoint inhibitors also show great promise for combination with oncolytic viruses. PD-1 and PD-L1 inhibitors block the association of the ligand (PD-L1) with its receptor (PD-1). Interaction of these cell surface proteins suppresses the immune response and is a mechanism that allows tumor cells to avoid immune surveillance. Several PD-1/PD-L1-based therapies for MM are currently being investigated in clinical trials [116]. Anti-PD-1/PD-L1 antibody therapy requires high levels of PD-L1 expression by tumor cells to be effective. Unfortunately, CD138^+^ cells from MM patients do not significantly overexpress PD-L1 compared to normal plasma cells [38]. This finding may explain, in part, why a phase I clinical trial using nivolumab found that patients with relapsed/refractory MM had little response to anti-PD-1 antibody treatment [117]. To overcome this problem, RV therapy was used to boost PD-L1 expression prior to treatment with anti-PD-L1 antibody. RV infection was shown to enhance expression of PD-L1 on both MM cell lines and CD138^+^ patient samples. In a mouse model of disseminated MM, RV/anti-PD-L1 antibody therapy significantly decreased tumor volumes and serum immunoglobulin levels while prolonging survival compared to either single agent therapy [38]. This is the first example of an oncolytic virus being combined with an immune checkpoint inhibitor to improve therapy in MM. Anti-PD-L1 therapy has also been successfully used with VSV-IFN-β-NIS in a mouse model of AML [118], suggesting that combining oncolytic viruses and immune checkpoint inhibitors may be a useful strategy for hematological malignancies.

Studies have demonstrated that MM cells are under intrinsic ER stress resulting from production of large amounts of immunoglobulin [119,120]. Consistent with this phenomenon, induction of ER stress due to the accumulation of undegraded ubiquitinated proteins is a major contributor to bortezomib’s anti-myeloma activity [120,121,122,123,124]. Therefore, agents with the ability to increase ER stress show strong anti-myeloma activity and augment bortezomib-mediated cell death. The accumulation of viral particles following treatment with RV, along with ubiquitin-conjugated protein accumulation in MM cells stimulated by bortezomib, promotes synergistic levels of ER stress and apoptosis [31]. RV and bortezomib combination therapy is currently under investigation in a phase I trial in patients with relapsed/refractory MM (Table 2).

## 4. Conclusions

Although significant progress has been made in the treatment of MM, it remains an incurable disease. New treatments options are urgently needed, particularly for patients that become resistant to conventional therapies. Oncolytic viruses are a new class of therapeutics that provide tumor-targeted therapy. Preclinical studies have identified several oncolytic viruses that are promising candidates for MM therapy. Three of these (RV, MV and VSV) have progressed to clinical trials. However, early clinical trials demonstrate that oncolytic viral therapy may be most impactful in combination with other anticancer agents. Future work should identify new strategies to enhance the efficacy of existing oncolytics. Specifically, treatment with immune checkpoint inhibitors have shown significant promise in combination with oncolytic viral therapy. Analysis of specimens from patients treated on clinical trials with antibodies that target PD-1 demonstrate that high basal expression of PD-L1 on tumor cells may be necessary to elicit significant clinical benefit [125]. This suggests that novel immune priming strategies, such as oncolytic viral therapy, that stimulate upregulation of PD-L1 on malignant cells could render agents that target the PD-L1/PD-1 axis significantly more effective for cancer patients with low PD-L1 expression, including patients with MM. Immune checkpoint inhibitor and oncolytic viral therapy combination clinical trials are currently being developed to investigate this promising therapeutic approach in patients with MM and other malignancies. The great potential that oncolytic virotherapies have shown makes this an exciting time for both researchers and patients with MM.

## Figures and Tables

**Table 1:** Oncolytic viruses currently being developed for multiple myeloma therapy.

	Reo	Measles	VSV	Vaccinia	Myxoma	Coxsackie	Adeno
**Genome**	dsRNA	ss(−)RNA	ss(−)RNA	dsDNA	dsDNA	ss(+)RNA	dsDNA
**Enveloped**	No	Yes	Yes	Yes	Yes	No	No
**Replication Site**	Cyto	Cyto	Cyto	Cyto	Cyto	Cyto	Nuc/cyto
**Receptors for Multiple Myeloma**	JAM-A	CD46	LDLRs	UK	UK	ICAM-1, DAF	UK
**Genetic Manipulation**	Difficult	Easy	Easy	Easy	Easy	Easy	Easy
**Combination Therapy**	BZ, LND, PMD, anti-PD-L1	CP	BZ, CP	NR	NR	NR	NR

Abbreviations: Cyto-cytoplasm; Nuc-nucleus; UK-unknown; BZ-bortezomib; LND-lenalidomide; PMD-pomalidomide; CP-cyclophosphamide; NR-none reported; VSV-Vesicular stomatitis virus.

**Table 2:** Clinical trials for oncolytic viruses in multiple myeloma.

Therapy	Phase	Combination Agents	Clinicaltrials.gov Identifier
**Reovirus (Reolysin)**	I	None	NCT01533194 [37]
	I	Lenalidomide or pomalidomide	NCT03015922
	I	Bortezomib + dexamethasone	NCT02514382
	I	Carfilzomib + dexamethasone	NCT02101944
**Measles (MV-NIS)**	I/II	±Cyclophosphamide	NCT00450814
	II	Cyclophosphamide	NCT02192775
	I	±Cyclophosphamide	NCT00450814
**VSV (VSV-IFNB-NIS)**	I	±Cyclophosphamide	NCT03017820
	I	±Cyclophosphamide	NCT00450814

## Abbreviations

The following abbreviations are used in this manuscript:

MM	multiple myeloma
MGUS	monoclonal gammopathy of unknown significance
AHSCT	autologous hematopoietic stem cell transplant
RV	reovirus
JAM-A	junctional adhesion molecule A
PKR	protein kinase R
MV	measles virus
SLAM	signaling lymphocyte activation molecule
IFN	interferon
NIS	sodium iodide symporter
VSV	vesicular stomatitis virus
LDLR	low density lipoprotein family of receptor
VV	vaccinia virus
TK	thymidine kinase
VGF	vaccinia growth factor
PBMC	peripheral blood mononuclear cell
MYXV	myxoma virus
GVHD	graft-versus-host disease
CV	coxsackievirus
AdV	adenovirus
